# Antibody–drug conjugates in urothelial carcinoma: scientometric analysis and clinical trials analysis

**DOI:** 10.3389/fonc.2024.1323366

**Published:** 2024-03-14

**Authors:** Meng Zhang, Yuanye Zuo, Siyi Chen, Yaonan Li, Yang Xing, Lei Yang, Hong Wang, Rui Guo

**Affiliations:** ^1^ Department of Clinical Laboratory, First Affiliated Hospital of Jilin University, Changchun, China; ^2^ Cancer Center, The First Hospital of Jilin University, Changchun, China

**Keywords:** scientometric analysis, clinical trials, urothelial carcinoma, antibody-drug conjugates, EV, SG, combination therapy

## Abstract

In 2020, bladder cancer, which commonly presents as urothelial carcinoma, became the 10th most common malignancy. For patients with metastatic urothelial carcinoma, the standard first-line treatment remains platinum-based chemotherapy, with immunotherapy serving as an alternative in cases of programmed death ligand 1 expression. However, treatment options become limited upon resistance to platinum and programmed death 1 or programmed death ligand 1 agents. Since the FDA’s approval of Enfortumab Vedotin and Sacituzumab Govitecan, the therapeutic landscape has expanded, heralding a shift towards antibody–drug conjugates as potential first-line therapies. Our review employed a robust scientometric approach to assess 475 publications on antibody–drug conjugates in urothelial carcinoma, revealing a surge in related studies since 2018, predominantly led by U.S. institutions. Moreover, 89 clinical trials were examined, with 36 in Phase II and 13 in Phase III, exploring antibody–drug conjugates as both monotherapies and in combination with other agents. Promisingly, novel targets like HER-2 and EpCAM exhibit substantial therapeutic potential. These findings affirm the increasing significance of antibody–drug conjugates in urothelial carcinoma treatment, transitioning them from posterior-line to frontline therapies. Future research is poised to focus on new therapeutic targets, combination therapy optimization, treatment personalization, exploration of double antibody-coupled drugs, and strategies to overcome drug resistance.

## 1 Introduction

In 2020, bladder cancer (BC) ranked as the tenth most common malignant neoplasm, accounting for an estimated 573,278 new cases and 212,536 mortalities globally (1). Urothelial carcinoma (UC) constitutes 90-95% of all BC cases, while neoplasms of the renal pelvis, ureter, and urethra remain infrequent (2). For patients with metastatic urothelial carcinoma (mUC), platinum-based chemotherapy serves as the cornerstone of first-line therapeutic modalities. Immunotherapy has emerged as a viable alternative for cisplatin-ineligible patients who express programmed death ligand 1 (PD-L1) (3). Systemic platinum-based chemotherapy, followed by maintenance therapy with avelumab, represents the sole first-line treatment for mUC proven to confer an overall survival benefit (4). However, therapeutic alternatives are markedly restricted for UC patients who are both platinum-ineligible and PD-L1-negative. Conventional chemotherapy and immune checkpoint inhibitors (ICIs) have not offered ideal long-term benefits in mUC before (5). In April 2023, the combination of enfortumab vedotin (EV) and pembrolizumab, demonstrating promising therapeutic effects in the EV-103 trial, received FDA approval for advanced urothelial carcinoma patients who are ineligible for cisplatin (6, 7). Subsequently, in December 2023, the FDA granted approval for this combination to treat locally advanced or metastatic urothelial cancer. This decision was based on the outcomes from the EV-302 trial, which showed a median overall survival (OS) that was double that of standard chemotherapy (8). Combination therapy involving nivolumab and gemcitabine-cisplatin has demonstrated significantly improved outcomes compared to gemcitabine-cisplatin monotherapy in patients with previously untreated advanced urothelial carcinoma (9). ADCs and combination therapy show broad and promising prospects in the treatment of UC.

As new anti-tumor drugs, antibody–drug conjugates(ADCs) have made significant advancements in the first-line or second-line therapy for UC in recent years. As the first clinical trial of ADCs in UC, AGS15E-13-1(NCT01963052) is a Phase I clinical trial studying the monotherapy dose of AGS15E in mUC, which initiated in but is yet to publish results. By now, the FDA has already approved EV and SG for the management of locally advanced or metastatic UC following ICIs and platinum-based therapies. Targeting Nectin-4, EV has demonstrated superior therapeutic efficacy in UC based on clinical trials, notably EV-301, which yielded a prolonged overall survival (12.88 vs. 8.97 months) and increased progression-free survival (PFS, 5.55 vs. 3.71 months) compared to traditional chemotherapy (10). SG exhibits considerable efficacy in cohort 1 of TROPHY-U-01, yielding a median objective response rate (ORR) of 27%, accompanied by a median progression-free survival (PFS) duration of 5.4 months and a median overall survival (OS) of 10.9 months (11).

Recently, ADCs have emerged as contenders to traditional first-line chemotherapy protocols in the management of UC. EV-302, a Phase III, two-arm trial, aims to assess the therapeutic effect of EV in conjunction with pembrolizumab as opposed to standard chemotherapy in treatment-naïve patients diagnosed with locally advanced or metastatic UC (12). RC48-C016, a Phase III trial, is designed to evaluate the therapeutic effect of RC48 in conjunction with JS001 as compared to standalone chemotherapy in untreated patients presenting with HER2-expressing, unresectable, locally advanced, or metastatic UC. There is a phase I/II trial (NCT04863885) assessing the first-line therapeutic effect of a combined regimen involving SG, ipilimumab, and nivolumab in cisplatin-ineligible mUC. These studies are ongoing. What is more, ADCs have explored the potential in the neoadjuvant and adjuvant therapy of UC. For instance, RG1122399(NCT05581589) is designed to assess the effectiveness of SG in the neoadjuvant interventions of non-muscle invasive bladder cancer. IUNU-UC-102 (NCT05016973) is a Phase II clinical trial estimating RC48 combined with Torialimab as neoadjuvant therapy for myometrial invasive bladder cancer. RC48-TA001 (NCT05356351) is another Phase II study targeting the neoadjuvant therapeutic potential of RC48 plus Torialimab in HER2-positive muscle-invasive bladder cancer.

Utilizing a multi-dimensional approach that integrates both scientometric data and clinical trials, this article elucidates the evolving landscape of ADCs in UC, thereby pinpointing key research trends and future directions. Bibliometric analysis offers a robust statistical examination of scholarly publications, facilitating the generation of network knowledge maps, identification of emerging trends, and highlighting of contemporary advancements in specific fields (13). Complementarily, the analysis of clinical trials furnishes crucial insights into the historical trajectory, current status, and prospective avenues for research and development within a field. This study undertook a rigorous scientometric examination of 475 publications pertaining to ADCs in UC, sourced from the Web of Science Core Collection (WoSCC) and published from 2005 to 2023. Analytic methodologies employed included the Web of Science (WoS) platform, Citespace, and VOS viewer. Within the realm of clinical trials, data were procured from ClinicalTrials.gov (n=63) and the International Clinical Trials Registry Platform (ICTRP; n=70). A total of 89 trials underwent comprehensive scrutiny. Both the scientometric evaluation and the clinical trial assessment corroborate a significant recent expansion in the field of ADCs for UC. Future research hotspots of ADCs are anticipated to include the discovery of new therapeutic targets, the optimization of combination therapies, and personalization of treatment regimens. Furthermore, delineating the mechanisms of drug resistance and investigating strategies to overcome such resistance are imperative for the advancement of ADCs in the therapeutic management of UC.

## 2 Materials and methods

### 2.1 Scientometric analysis

Web of Science Core Collection (WoSCC) is one of the fundamental data sources for both Citespace (6.1.R6) and VOSviewer (1.6.20). The source of this scientometric analysis is the Science Citation Index Expanded in WoSCC. The retrieval terms were chosen based on the Medical Subject Headings (Mesh) database: TS=(“bladder cancer” OR “bladder carcinoma” OR “bladder tumor” OR “Bladder Neoplasm” OR “cancer of the bladder” OR “CARCINOMA OF BLADDER” OR “urothelial carcinoma” OR “urothelial cancer” OR “urothelial tumor” OR “urothelial neoplasm” OR “upper urinary tract urothelial carcinoma” OR “upper urinary tract urothelial cancer” OR “upper urinary tract urothelial neoplasm” OR “upper urinary tract urothelial tumor” OR “carcinoma of the bladder” OR “Neoplasm of Urinary Bladder” OR “Tumor of the Urinary Bladder” OR “Tumor of the Bladder” OR “carcinoma of the bladder” OR “Carcinoma of Urinary Bladder” OR “Carcinoma of the Urinary Bladder” OR “carcinoma of the urothelial bladder” OR “Carcinoma of the upper urinary tract urothelial” OR “cancer of the upper urinary tract urothelial” OR “tumor of the upper urinary tract urothelial” OR “neoplasm of the upper urinary tract urothelial”) AND TS=(“antibody-drug conjugate” OR “antibody drug conjugate” OR “T-DM1” OR “RC48” OR “claudin18.2” OR “DS-8201” OR “DS8201” OR “DS-8201a” OR “Enfortumab vedotin” OR “claudin” OR “Oportuzumab monatox” OR “ASG-15ME” OR “RC-48” OR “Disitamab Vedotin” OR “Trastuzumab deruxtecan” OR “Gemtuzumab ozogamicin” OR “Brentuximab vedotin” OR “Inotuzumab ozogamicin” OR “Polatuzumab vedotin” OR “Trastuzumab emtansine” OR “sacituzumab govitecan”). The years searched were from 2005 to 2023. The searching process was conducted on Dec. 24, 2023. Finally, the information of 475 documents was downloaded in the form of Plain Text File and Tab delimited file.

Web of Science (WoS). The WoS platform provides the annual distribution of publications and citations.

Citespace (6.1.R6). The 475 search results were downloaded from WoSCC in the plain text file form. In this study, Citespace was used to provide analysis of study countries and institutions.

VOS viewer (1.6.20). The 475 search results were downloaded from WoSCC in the tab delimited file form. VOS viewer was used to analyze keywords of this field.

Bibliometrics refers to a set of methods used for quantitatively analyzing scientific literature. This approach involves collecting and evaluating data from publications over a certain period. Through this analysis, bibliometrics provides essential insights into the level of scientific output, patterns of behavior, and progress in a particular field of research (14). The objective of this study was to employ bibliometric analysis and discuss the results in a narrative review pattern to elucidate the current status and emergent trends in the field of ADCs of UC.

### 2.2 Clinical trials

ClinicalTrials.gov (https://www.clinicaltrials.gov) and WHO ICTRP (https://trialsearch.who.int) are the source of clinical trials in this study. The searching strategies in ClinicalTrials.gov/ICTRP were: 1) Condition or Disease/Condition = (“Urothelial Cancer” OR “Urothelial Carcinoma” OR “Urothelial Neoplasm” OR “Urothelial Carcinoma Bladder” OR “Urothelial Carcinoma Ureter” OR “Urothelial Carcinoma Urethra” OR “Urothelial Cancer of Renal Pelvis”), 2) Other terms/Innervation = (“antibody-drug conjugate” OR “antibody drug conjugate” OR “T-DM1” OR “RC48” OR “claudin18.2” OR “DS-8201” OR “DS8201” OR “DS-8201a” OR “Enfortumab vedotin” OR “claudin” OR “Oportuzumab monatox” OR “ASG-15ME” OR “RC-48” OR “Disitamab Vedotin” OR “Trastuzumab deruxtecan” OR “Gemtuzumab ozogamicin” OR “Brentuximab vedotin” OR “Inotuzumab ozogamicin” OR “Polatuzumab vedotin” OR “Trastuzumab emtansine” OR “sacituzumab govitecan”). There were 63 trials from ClinicalTrials.gov and 70 trials from ICTRP. After duplicating by Trial IDs and reviewing each trial, there were 89 clinical trials incorporated into this study. The time of searching and handling the data was Dec. 2023. The specific and detailed information of 89 clinical trials was recognized, including start year, intervention, phases (phase I, phase I/II, phase II, phase III, and others) and some important drugs (EV, SG, RC48, Pembrolizumab and so on). After the statistical process of all clinical trials, the corresponding analysis was made.

### 2.3 Modify

ChatGPT (GPT-4 version, a multimodal model, from OpenAI) was applied to modify the language of this article.

## 3 Results

### 3.1 Scientometric analysis

#### 3.1.1 Distribution of publications and citations by year

**Figure 1:**
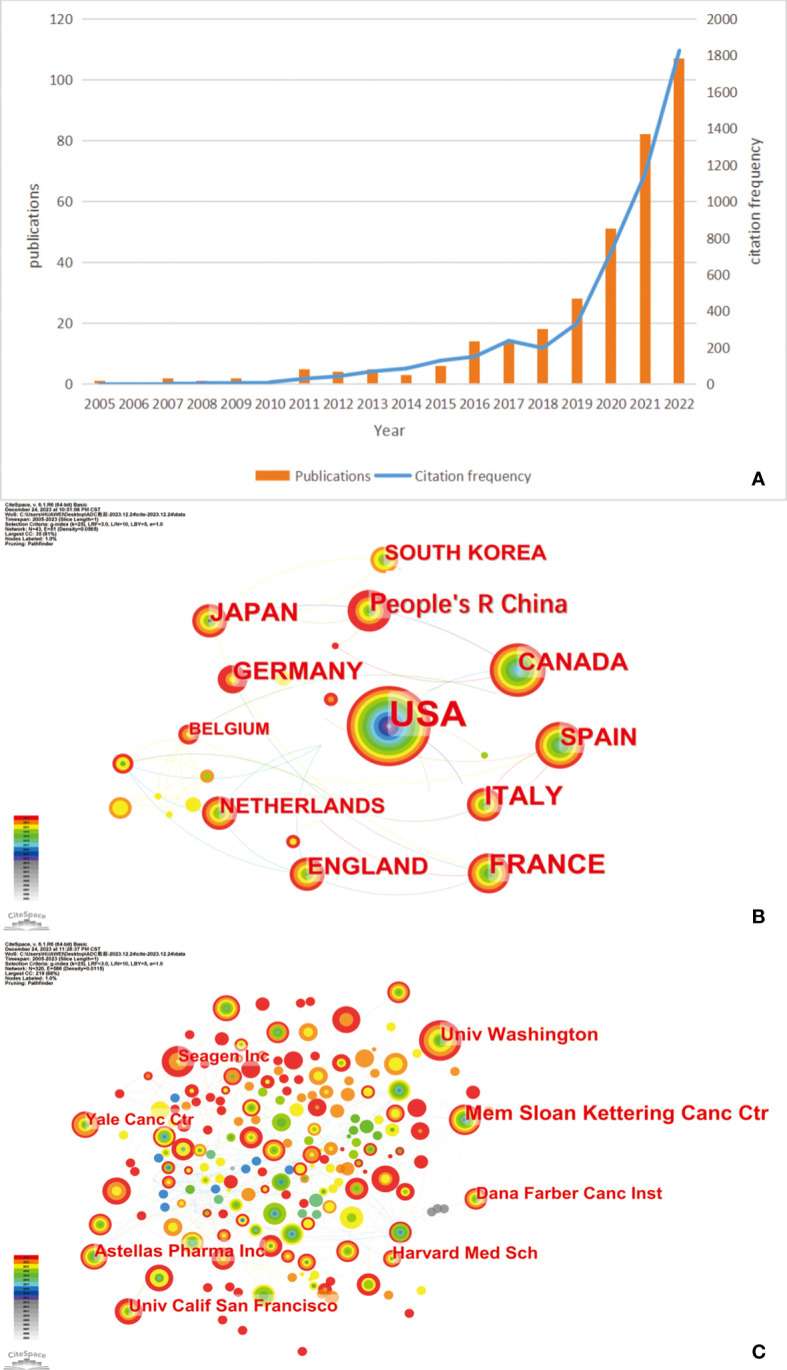
**(A)** The annual distribution of publications and citation frequency related to antibody–drug conjugates (ADCs) in urothelial carcinoma (UC). **(B)** A network map showing the top 12 countries with the most publications in ADCs in UC. **(C)** The top 8 institutions regarding ADCs in UC. Created with CiteSpace.

Based on the specialized searching terms in WoSCC, this study encompasses a corpus of 475 files and 4314 cited documents. Cumulatively, these cited documents have garnered 6950 citations, yielding an average citation frequency of 14.63 per article. The H-index is 42, signifying that a total of 42 scholarly manuscripts have garnered an excess of 42 citations each. **Figure 1A** delineates the annual distribution of publication and citation frequency. As **Figure 1A** shows, there is a general growth trend from 2005 to 2022. From 2005 to 2018, publication volume increases slowly. After 2018, both publication counts and the number of citations rises sharply.

#### 3.1.2 Related countries and institutions

Based on the data from WoSCC, we used CiteSpace to analyze the country and institution in this field and displayed the results in **Figure 1B**. As **Figure 1B** shows, the USA emerges as the preeminent contributor, distinguished not only by its early initiation into the field but also by its extensive collaborations with other nations. As the data shows, the USA leads in the number of published articles (n=264, 34.24%), followed by France (n = 61,7.91%) and Italy (n = 54; 7.00%). **Figure 1C** illustrates the contributing countries with respect to ADCs in UC. We collected the top 10 institutions with the most publications in this field and listed them in **Table 1**. Memorial Sloan Kettering Cancer Center is the institution with the highest number of published papers, nearly 63 (19.33%), followed by the University of Washington (n = 43; 13.19%) and Astellas Pharma Inc (n = 33; 10.12%).

**Table 1:** The top 10 institutions with most publications.

Institution	Year	Count	Percentage
Mem Sloan Kettering Canc Ctr	2016	63	19.33%
Univ Washington	2019	43	13.19%
Astellas Pharma Inc	2017	33	10.12%
Univ Calif San Francisco	2015	31	9.51%
Harvard Med Sch	2017	31	9.51%
Dana Farber Canc Inst	2018	29	8.90%
Seagen Inc	2021	28	8.59%
Yale Canc Ctr	2019	24	7.36%
Univ Chicago	2011	22	6.75%
Stanford Univ	2011	22	6.75%

#### References

3.1.3

Co-citation analysis can reflect the academic research direction and academic cross-development. We selected the 10 most cited articles to make **Table 2** for further analysis. The article named “Enfortumab Vedotin in Previously Treated Advanced Urothelial Carcinoma” is cited most, with 337 citations, and was published in the NEW ENGLAND JOURNAL OF MEDICINE in 2021. The second most cited article is “Pivotal Trial of Enfortumab Vedotin in Urothelial Carcinoma After Platinum and Anti-Programmed Death 1/Programmed Death Ligand 1 Therapy”, followed by “Trastuzumab duocarmazine in locally advanced and metastatic solid tumours and HER2-expressing breast cancer: a phase 1 dose-escalation and dose-expansion study”.

**Table 2:** Top 10 highly cited documents related to the antibody-conjugated drugs in urothelial carcinoma according to Web of Science database.

Year	Title	Type	First author	Journal	IF	JCR	Co-citation
2021	Enfortumab Vedotin in Previously Treated Advanced Urothelial Carcinoma	Article	Powles, T	NEW ENGLAND JOURNAL OF MEDICINE	158.5	Q1	337
2019	Pivotal Trial of Enfortumab Vedotin in Urothelial Carcinoma After Platinum and Anti-Programmed Death 1/Programmed Death Ligand 1 Therapy	Article	Rosenberg, JE	JOURNAL OF CLINICAL ONCOLOGY	45.3	Q1	314
2019	Trastuzumab duocarmazine in locally advanced and metastatic solid tumours and HER2-expressing breast cancer: a phase 1 dose-escalation and dose-expansion study	Article	Banerji, U	LANCET ONCOLOGY	51.1	Q1	285
2011	Immunohistochemical Diagnosis of Renal Neoplasms	Article	Truong, LD	ARCHIVES OF PATHOLOGY & LABORATORY MEDICINE	4.6	Q1	237
2014	Intrinsic basal and luminal subtypes of muscle-invasive bladder cancer	Review	Choi, W	NATURE REVIEWS UROLOGY	15.3	Q1	213
2021	TROPHY-U-01: A Phase II Open-Label Study of Sacituzumab Govitecan in Patients With Metastatic Urothelial Carcinoma Progressing After Platinum-Based Chemotherapy and Checkpoint Inhibitors	Article	Tagawa, ST	JOURNAL OF CLINICAL ONCOLOGY	45.3	Q1	180
2017	Impact of Molecular Subtypes in Muscle-invasive Bladder Cancer on Predicting Response and Survival after Neoadjuvant Chemotherapy	Article	Seiler, R	EUROPEAN UROLOGY	23.4	Q1	165
2019	Management of metastatic bladder cancer	Review	Nadal, R	CANCER TREATMENT REVIEWS	23.4	Q1	160
2014	HER2 aberrations in cancer: Implications for therapy	Review	Yan, M	CANCER TREATMENT REVIEWS	11.8	Q1	159
2016	Claudin-low bladder tumors are immune infiltrated and actively immune suppressed	Article	Kardos, J	JCI INSIGHT	8	Q1	147

#### 3.1.4 Co-occurrence analysis of keywords

**Figure 2:**
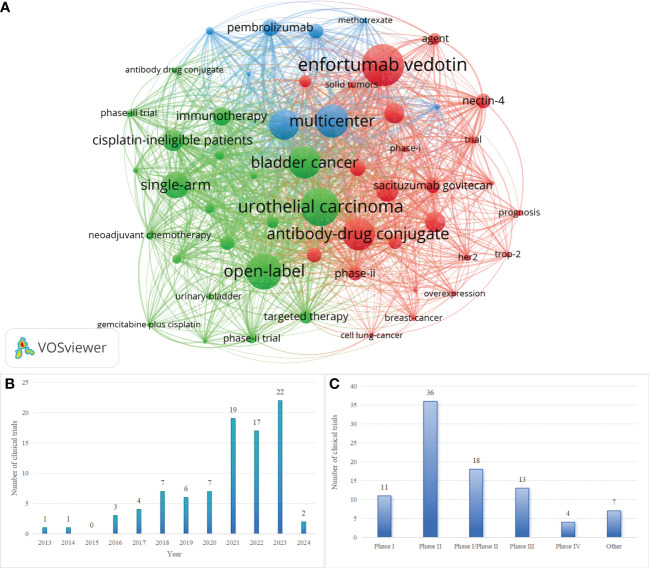
**(A)** Keyword co-occurrence map involved in ADCs in UC. **(B)** Annual Distribution of Clinical Trials Related to ADCs in UC. **(C)** Distributions of Phases for Clinical Trials Related to ADCs in UC. Created with VOSviewer.

Keywords show research hotspots in a certain field. Keyword co-occurrence analysis is helpful to evaluate the research hotspots and the relationship of topics in the discipline. After processing the keywords, which appear less than 10 times, 53 keywords are visualized through VOSviewer. **Figure 2A** shows the bibliometric analysis of keywords, of ADCs in UC. The most frequent keyword is Enfortumab Vedotin, whose node is the biggest at 94. Following this are urothelial carcinoma (n=86) and open-label(n=80) in turn.

### 3.2 Clinical trials

#### 3.2.1 Development and current status of relevant clinical trials

We retrieved 89 clinical trials related to ADCs in UC from ClinicalTrials.gov (https://www.clinicaltrials.gov) and WHO ICTRP (https://trialsearch.who.int). By analyzing the clinical investigations, we found that the first clinical trials of ADCs in UC started in 2013 (NCT01963052): “A Phase 1 Study of the Safety and Pharmacokinetics of Escalating Doses of AGS15E Given as Monotherapy in Subjects With Metastatic Urothelial Cancer”. Over the subsequent four years, the quantity of related clinical trials remained low. A period of stability in trial numbers was observed from 2018 to 2020, followed by a significant surge, and then kept a high level until 2023. **Figure 2B** shows the annual distribution of clinical trials. Of the 89 clinical trials, there are 7 trials with results. Both **Figure 1A** and **Figure 2B** represent the development of ADCs in UC and the analysis states the anticipated potential of this field.

**Figure 2C** shows the phases of related clinical trials. Of the 89 clinical trials, PhaseII(n=36) are the majority, Phase I(n=11) and PhaseIII(n=13) are at a similar level, and 18 clinical trials are in Phase I |Phase II. The trials of PhaseIII represent the leading ADCs in UC and they are displayed in **Table 3**.

**Table 3:** All the relevant clinical trials in Phase III.

No.	Trial ID	Start Date	Result	Title
1	NCT04223856	Mar.2020	no	Enfortumab Vedotin and Pembrolizumab vs. Chemotherapy Alone in Untreated Locally Advanced or Metastatic Urothelial Cancer
2	NCT03474107	Jun.2018	Has Results	A Study to Evaluate Enfortumab Vedotin Versus (vs) Chemotherapy in Subjects With Previously Treated Locally Advanced or Metastatic Urothelial Cancer (EV-301)
3	NCT05302284	Jun.2022	no	A Study of RC48-ADC Combined With Toripalimab For First-line Treatment of Urothelial Carcinoma
4	NCT05754853	Apr. 2023	no	A Study of MRG002 Versus Investigator’s Choice of Chemotherapy in the Treatment of Patients With HER2-positive Unresectable Advanced or Metastatic Urothelial Cancer
5	NCT04527991	Jan.2021	no	Study of Sacituzumab Govitecan-hziy (IMMU-132) Versus Treatment of Physician’s Choice in Participants With Metastatic or Locally Advanced Unresectable Urothelial Cancer
6	EUCTR2020-005452-38-AT	June.2021	no	Treatment combination of Durvalumab, Tremelimumab, Enfortumab Vedotin in patients with muscle invasive bladder cancer ineligible to cisplatin
7	EUCTR2020-005452-38-DE	Jun.2022	no	Treatment combination of Durvalumab, Tremelimumab, Enfortumab Vedotin in patients with muscle invasive bladder cancer ineligible to cisplatin or Who Refuse Cisplatin
8	NCT03568318	Jun.2018	Has results	A Study to Evaluate Upadacitinib in Combination With Topical Corticosteroids in Adolescent and Adult Participants With Moderate to Severe Atopic Dermatitis
9	EUCTR2020-003106-31-DE	Dec.2020	no	Phase 3 Trial of Perioperative EV and Pembro vs. Chemo in Cis-E MIBC
10	EUCTR2017-003344-21-DE	Apr.2018	Has Results	An Open-Label, Randomized Phase 3 Study to Evaluate Enfortumab Vedotin vs Chemotherapy in Subjects with Previously Treated Locally Advanced or Metastatic Urothelial Cancer (EV-301)
11	JPRN-jRCT2031200284	Jan.2021	no	Enfortumab vedotin and pembrolizumab vs. chemotherapy alone in untreated locally advanced or metastatic urothelial cancer
12	NCT05911295	Sep.2023	no	Disitamab Vedotin With Pembrolizumab vs Chemotherapy in Previously Untreated Urothelial Cancer Expressing HER2
13	ChiCTR2300069410	March.2023	no	An open-label, randomized, controlled phase 3 study of enfortumab vedotin in combination with pembrolizumab versus chemotherapy alone in previously untreated locally advanced or metastatic urothelial cancer

**Figure 3:**
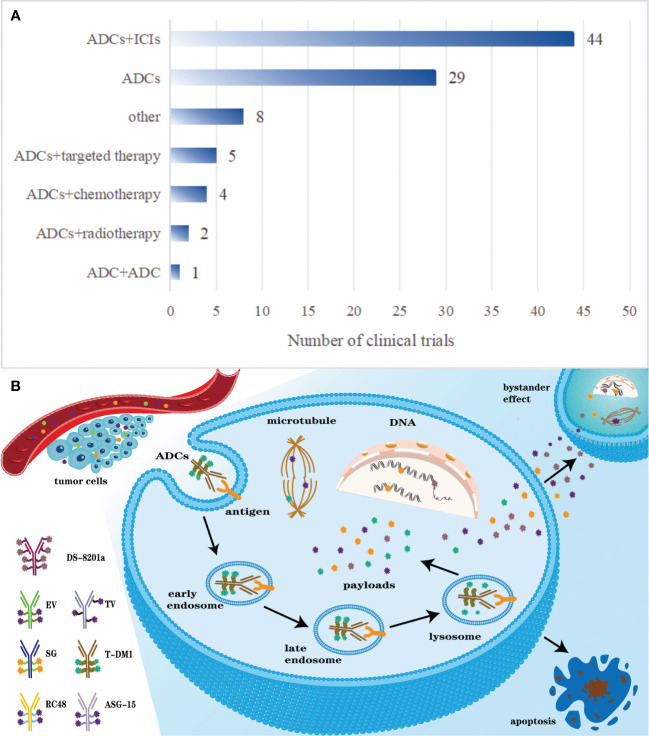
**(A)** The distribution of some common therapies related to ADCs. **(B)** Pharmacodynamics of ADCs after intravenous administration.

An in-depth analysis of 89 clinical trials revealed 29 trials focusing on ADCs monotherapy, 52 trials exploring combination therapies based on ADCs, and 9 trials investigating other approaches. Among the 52 trials on combination therapy, the most frequent regimen combines ADCs with ICIs, accounting for 44 trials. ADCs in conjunction with chemotherapy (n=4) and targeted therapy (n=5) exhibit comparable levels of research interest. Two trials are dedicated to the combination of ADCs and radiotherapy, while a singular trial explores the co-use of two different ADCs. Notably, both NCT03869190 and NCT03288545 investigate two combinations: ADCs with ICIs and ADCs with chemotherapy. NCT05489211 is unique in studying three combinations: ADCs with ICIs, targeted therapy, and chemotherapy. **Figure 3A** illustrates the distribution of these treatment modalities.

#### 3.2.2 Some important ADCs or drugs in relevant clinical trials

In 2019, EV, the inaugural ADC targeting Nectin-4, received FDA approval for locally advanced and metastatic UC therapy. Among the 89 clinical trials concerning ADCs in UC, the clinical trials include 7 Phase I, 10 Phase II, 8 Phase III, 6 Phase I/Phase II,1 Phase IV, and 6 others. 12 trials are dedicated to exploring EV as a monotherapy, while 22 are designed to assess combination therapies based on EV. And there are 4 clinical trials concerning the side effects and life quality of EV. EV combined with Pembrolizumab is the majority(n=15) among the research on combination therapy based on EV. Of the 89 clinical trials, NCT03474107, NCT03219333, NCT04995419, NCT03606174, and EUCTR2017-003344-21-DE have already yielded promising results. The first clinical trial of EV in UC started in 2014 and the yearly number of related clinical trials maintained a low level until 2020. In 2021, it reached a peak with 11 trials.

Disitamab Vedotin(RC48) represents a novel ADC targeting HER2 and is constructed of Hertuzumab conjugated to monomethyl auristatin E (MMAE) via a cleavable linker. Among the 89 clinical trials, there are 20 clinical trials related to RC48, primarily in Phase II (n=12). The majority of clinical trials investigate combination therapies incorporating RC48 (n=17). The first clinical trial started in 2017 and most experiments started in 2022(n=8). To date, none of these 20 trials have reported results.

Sacituzumab Govitecan (SG) is an ADC targeting TROP-2 that received accelerated FDA approval for advanced UC patients with prior exposure to platinum-based chemotherapy and PD-1/PD-L1 inhibitors. Of the 89 clinical trials related to ADCs in UC, there are 16 clinical trials related to SG, which are predominantly in Phase II (n=8). The inaugural clinical trial started in 2018, titled “Study of Sacituzumab Govitecan in Participants With Urothelial Cancer That Cannot Be Removed or Has Spread”, and 7 clinical trials started in 2021. Thus far, all SG-related trials are awaiting results.

As a notable ICI, pembrolizumab appears in 22 clinical trials related to ADCs in UC. Of the 22 clinical trials, Phase II (n=11) and Phase III (n=5) investigations predominate. It is mainly combined with other drugs in the 22 clinical trials. The first clinical trial involving pembrolizumab was conducted in 2017, the title of which is “A Study of Enfortumab Vedotin Alone or With Other Therapies for Treatment of Urothelial Cancer”. A notable uptick was observed in 2023, with 8 new clinical trials initiated.

## 4 Discussions

### 4.1 ADCs

#### 4.1.1 The development of ADCs

The conceptual underpinning of antibody-drug conjugates (ADCs) traces its lineage to Paul Ehrlich’s early 20th-century notion of the “magic bullet”. This concept posited that toxins could be selectively delivered to cancer cells without damaging normal tissue if mounted on a precise carrier. In the initial stages, ADCs, exemplified by BR96-doxorubicin, predominantly consisted of a traditional chemotherapy drug linked to a mouse-derived antibody via a non-cleavable linker, resulting in substantial immunogenicity (15, 16). With technological advancements, humanized monoclonal antibodies have superseded mouse-derived antibodies, significantly enhancing both efficacy and safety.

##### 4.1.1.1 The first-generation ADCs

With the introduction of more potent cytotoxic drugs and reduced immunogenicity, the first-generation ADCs, such as gemtuzumab ozogamicin and inotuzumab ozogamicin, have been approved for the market. These ADCs utilize IgG4 and are conjugated to calicheamicin, a potent cytotoxic agent, through acid-labile linkers (17, 18). Gemtuzumab ozogamicin, as the first ADCs approved by the FDA, was indicated for the treatment of first-relapsed acute myeloid leukemia (AML) in patients aged 60 years or older who are CD33 positive and unsuitable for cytotoxic chemotherapy (19). However, it was withdrawn in 2010 due to concerns regarding its toxicity and efficacy. In 2017, the FDA reapproved gemtuzumab ozogamicin for monotherapy in R/R CD33-positive AML patients, utilizing a regimen with lower-dose fractionation (20). Inotuzumab ozogamicin, a humanized CD-22–targeting ADCs, is utilized as a monotherapy in the treatment of adult patients with relapsed/refractory B-cell acute lymphoblastic leukemia (21). First-generation ADCs have demonstrated superior therapeutic effects; however, they are also associated with several significant issues. These include uncontrolled release, off-target toxicity, short half-life, rapid clearance, and immunogenicity, all of which require further improvement (22–24).

##### 4.1.1.2 The second-generation ADCs

Building upon the foundation of first-generation ADCs, the second-generation ADCs have improved by optimizing monoclonal antibody isoforms, cytotoxic payloads, and linkers. These advancements involve selecting more potent cytotoxic agents and IgG1, which are better suited for small molecule payloads and exhibit enhanced targeting capabilities for cancer cells (25, 26). Examples of second-generation ADCs include brentuximab vedotin and trastuzumab emtansine.

Brentuximab vedotin, a potent ADCs targeting CD30, consists of IgG1 antibodies linked to the payload MMAE via protease cleavable ligands (27). In August 2011, the FDA approved Brentuximab vedotin for treating patients with Hodgkin lymphoma and anaplastic large cell lymphoma (ALCL) (28). Furthermore, in March 2018, the FDA approved the coadministration of Brentuximab vedotin with chemotherapy agents (doxorubicin, dacarbazine, and vinblastine) for the treatment of adult patients with previously untreated stage III or IV classical Hodgkin lymphoma (29). Trastuzumab emtansine (T-DM1) received approval from the FDA and the EU in 2013 for the treatment of HER2-positive breast cancer (30).

Overall, second-generation ADCs demonstrate improved clinical efficacy and safety. However, there are still several unmet needs, including limited therapeutic windows due to off-target toxicity, and issues of aggregation or rapid clearance in ADCs with high drug-to-antibody ratio (DAR). This indicates substantial potential for development and optimization in third-generation ADCs.

##### 4.1.1.3 The third-generation ADCs

Third-generation ADCs, exemplified by polatuzumab vedotin, enfortumab vedotin, fam-trastuzumab deruxtecan, and other subsequently approved ADCs, employ fully humanized antibodies instead of chimeric antibodies to reduce immunogenicity. These third-generation ADCs exhibit lower toxicity and higher anticancer activity, along with enhanced stability, thereby providing patients with more effective anticancer therapy (31–33).

Polatuzumab vedotin, a CD79b-targeted ADC, delivers monomethyl auristatin E (MMAE), a microtubule inhibitor. In 2019, it was approved in combination with bendamustine and a rituximab product for adult patients with relapsed or refractory diffuse large B-cell lymphoma (DLBCL), not otherwise specified, following at least two prior therapies. Trastuzumab deruxtecan, a conjugate of a HER2-directed antibody and a DNA topoisomerase I inhibitor, received approval in the USA based primarily on the phase 2 DESTINY-Breast01 trial. In 2019, EV was approved by the FDA for patients with locally advanced or metastatic UC who are ineligible for platinum-based therapies and ICIs. Then, in April 2023, the FDA approved the combination of EV and pembrolizumab for patients with advanced urothelial carcinoma who are ineligible for cisplatin.

In recent years, ADCs in many fields have flourished. So far, hundreds of ADCs have been used in clinical trials, of which 15 have been approved by the FDA, the European Medicines Agency (EMA), and/or other government agencies and have been put on the market for the treatment of hematological malignancies and solid tumors (34).

#### 4.1.2 The metabolism mechanism of ADCs

Comprising monoclonal antibody, cytotoxic small-molecule drug, and linker, ADCs amalgamate a potent cytotoxic efficacy of conventional chemotherapeutic agents with the targeted specificity conferred by antibodies. Administered intravenously, ADCs enter blood and distribute to targeted tumor tissue relying on the specific recognition of monoclonal antibodies and antigens that specifically overexpress or uniquely present on the tumor cell membrane. Upon antigen binding, ADCs undergo internalization into the cell through receptor-mediated endocytosis, initially entering early endosomes. These early endosomes subsequently mature into late endosomes prior to lysosomal fusion (19). ADCs with cleavable linkers are cleaved within endosomes or lysosomes, while those with non-cleavable linkers are processed only in lysosomes, leading to payload release and intracellular distribution (35, 36). The released payloads predominantly target nuclear and microsomal proteins, inhibiting tumor growth (22). The payloads of some ADCs with cleavable linkers could go through cell membrane and induce surrounding cells to go through apoptosis, which is called the bystander effect (37). The bystander effect can locally produce strong toxic effects on surrounding cells, which correlates with the antigen expression levels (38). Notably, preclinical models have elucidated the presence of a bystander effect associated with Sacituzumab Govitecan (SG), Enfortumab Vedotin (EV) and Tisotumab Vedotin (TV) (39, 40). **Figure 3B** elucidates the pharmacodynamics of ADCs post-intravenous administration.

#### 4.1.3 Composition of ADCs

ADCs have recently emerged as a prominent therapeutic agent in cancer treatment, providing targeted delivery combined with potent cytotoxic agents. The three primary components of an ADC—monoclonal antibody, payload, and linker—play crucial roles in determining its efficacy and safety.

##### 4.1.3.1 Antibody

ADCs utilize antibodies to specifically target antigens present on tumor cells. The ideal antigens for ADC antibody binding should be predominantly, if not exclusively, expressed on tumor cells and minimally or not at all on normal tissues, while also being non-secreted (41, 42). In the initial phase of ADC development, mouse-derived antibodies with high immunogenicity were commonly used, leading to a notable failure rate (43). Currently, most ADCs employ fully humanized antibodies, which significantly reduce immunogenicity, and a smaller proportion utilizes chimeric antibodies (15). Humanized or human monoclonal antibodies are not only expected to possess high specificity and efficient binding and internalization capabilities towards target antigens but should also demonstrate minimal immunogenicity, maintain prolonged plasma half-life, exhibit reduced cross-reactivity, and facilitate easy conjugation with small molecules (44, 45).

Immunoglobulin G (IgG), comprising four subtypes - IgG1, IgG2, IgG3, and IgG4 - is the most prevalent antibody utilized in ADCs (46). IgG1, notable for its abundance and high Fc receptor binding affinity, is the most commonly used subtype in ADCs. It induces strong effector functions with potent anti-cancer activities, such as antibody-dependent cell-mediated cytotoxicity (ADCC), antibody-dependent phagocytosis (ADCP), and complement-dependent cytotoxicity (CDC) (47) IgG2, however, tends to form dimers and aggregates *in vivo*, leading to reduced ADC drug concentrations (48) IgG3, with its notably short half-life of approximately 7 days, diminishes the therapeutic efficacy of ADCs and does not enhance Fc-mediated effector functions (49, 50). IgG4 can induce ADCP, but it is characterized by Fab-arm exchange, which results in decreased efficacy and impaired targeting (51, 52).

The dissociation constant (Kd) and molecular weight of the antibody are critical factors influencing the efficacy of ADCs. Kd, in particular, plays a pivotal role in the internalization of ADCs into cancer cells; a lower Kd indicates a higher binding affinity between the antibody and the neonatal Fc receptor (FcRn) on the tumor cell surface, which can paradoxically lead to reduced efficacy. Therefore, a higher Kd is desirable for effective penetration and uniform distribution across tumor cells (53). In the treatment of solid tumors, miniaturized antibodies maintain high affinity and specificity, and their smaller size facilitates easier penetration through blood vessels into the tumor tissue, significantly enhancing their efficacy in targeting and eradicating solid tumors. However, the large molecular weight of IgG antibodies (approximately 150 kDa) poses challenges in penetrating the blood capillary and tumor matrix (54). Consequently, researchers have attempted to miniaturize antibodies by removing the Fc segment to enhance their effectiveness. However, it has been observed that such miniaturized antibodies exhibit a reduced half-life *in vivo* (55). The process of miniaturizing antibodies is complex and multifaceted, necessitating careful consideration of various factors.

##### 4.1.3.2 Payloads

The cytotoxic payload in ADCs acts as the ‘warhead’, exerting cytotoxic effects post-internalization into cancer cells. Owing to the lysosomal barrier and the complexity of the tumor microenvironment, only approximately 2% of ADCs reach their targeted tumor sites following intravenous administration. Consequently, compounds used as ADCs payloads must possess high potency (22, 35). What is more, these payloads must possess potent cytotoxicity and demonstrate high stability, modifiability, hydrophobicity, and strong membrane permeability (56). The first-generation ADCs incorporated classical chemotherapy agents like doxorubicin and methotrexate, leveraging their well-established toxicity profiles (57). Given their strong target-specific toxicity, immunotherapeutic drugs have also become suitable candidates for ADCs payloads. Currently, ADCs payloads are primarily classified into two categories: potent tubulin inhibitors and DNA-damaging agents (58).

Tubulin inhibitors, which function during cell mitosis, encompass both tubulin polymerization promoters and inhibitors that disrupt microtubule-dependent mitosis (59). Tubulin polymerization promoters specifically target the β-subunits of the tubulin dimer, disrupting microtubule growth. These promoters are exemplified by auristatin derivatives, namely monomethyl auristatin E (MMAE) and monomethyl auristatin F (MMAF) (60, 61). Of the 15 approved ADCs, 5 utilize MMAE/MMAF as payloads. Conversely, tubulin polymerization inhibitors prevent the formation of mature microtubules by blocking the polymerization of the tubulin dimer. Maytansinoid derivatives like DM1 and DM4 are typical examples of such inhibitors (37). Ado-trastuzumab emtansine, approved by the FDA in 2013, was the inaugural ADC drug to be conjugated with maytansinoid derivatives.

The IC50 values of DNA-damaging agents can reach the picomolar level, making ADCs conjugated with these agents occasionally more effective than tubulin inhibitors and potentially effective irrespective of cell cycle stages. The primary mechanisms of action for DNA-damaging agents include: (i) inducing DNA double-strand breaks, as seen with calicheamicins; (ii) facilitating DNA alkylation, exemplified by duocarmycins; (iii) causing DNA intercalation, such as with topoisomerase I inhibitors; and (iv) promoting DNA crosslinking, as observed with pyrrolobenzodiazepines (PBD) (62–64).

In addition to traditional cytotoxins, ADC design is now increasingly incorporating payloads with novel mechanisms, including Toll-like receptor (TLR) agonists and stimulators of interferon genes (STING) agonists (36, 38). Furthermore, toxins exhibiting multiple mechanisms of action, carrying various loads simultaneously, immune-stimulating payloads, and radioactive isotopes are also being explored.

##### 4.1.3.3 Linker

The linker in ADCs functions as a vital bridge between the payload and the antibody, playing a key role in their stability and efficacy. An ideal linker should possess high water solubility, preventing both aggregation of the ADC and premature payload release in systemic circulation (39). Regarding intracellular stability and degradation patterns, linkers are classified into two distinct categories: cleavable and non-cleavable (40). Cleavable linkers exhibit sensitivity to intracellular environmental conditions and could be cleaved by acid, protease, a reducing substance, and so on (40). Typically, these linkers, including hydrazone bonds, disulfide bonds, and polypeptides, are cleaved within endosomes or lysosomes. In contrast, non-cleavable linkers undergo cleavage only within lysosomes, leading to the degradation of their associated payloads into amino acids (65). Significantly, non-cleavable linkers offer the benefits of an enhanced therapeutic index and reduced off-target toxicity (66). While each strategy presents its own advantages and drawbacks, the optimal linker and conjugation chemistry must be precisely tailored to align with the specific properties of the antibody, the drug molecule, and the disease profile being targeted.

###### 4.1.3.3.1 Non-cleavable linkers

Non-cleavable linkers in ADCs necessitate monoclonal antibody (mAb) degradation within the lysosome following ADCs internalization to release the active drug. A diverse range of non-cleavable alkyl and polymeric linkers have been investigated in ADCs development, including MCC amine-to-sulfhydryl bifunctional cross-linker, notably utilized in T-DM1 (25). The primary advantage of using non-cleavable linkers lies in their enhanced plasma stability compared to several cleavable linkers. Although non-cleavable linkers exhibit a limited ‘bystander’ effect, their resistance to cleavage outside target cells notably enhances the specificity of drug release (67).

###### 4.1.3.3.2 Chemically labile linkers

Cleavable linkers, notably acid-sensitive ones like hydrazones and silyl ethers, are prevalent in the ADCs clinical pipeline (68). Hydrazones are straightforward to synthesize, yet they exhibit selective cleavage under acidic conditions (69). However, given that acidic conditions are commonly found in various parts of the body, there is an increased likelihood of non-specific drug release. The first-generation ADC, gemtuzumab ozogamicin, which utilized a hydrazone linker, was withdrawn from the US market in 2010. This decision was partly due to the toxicities associated with the poor plasma stability of the hydrazone (70). Experimental findings indicate that drugs linked via disulfide bonds typically undergo initial release through antibody-mediated protein hydrolysis, followed by subsequent release as active metabolites via disulfide bond exchange or reduction (71). Subsequently, methylated drug metabolites are able to diffuse through lipid membranes to reach their target action sites.

###### 4.1.3.3.3 Enzymatically cleavable linkers

Enzymatically cleavable linkers are characterized by their superior plasma stability. A more controlled drug release can be achieved by attaching the drug to an antibody using a peptide linkage. This allows for the specific cleavage of the free drug from its carrier by lysosomal proteases, which are present in higher concentrations in certain tumor tissues (72). Peptidic bonds are anticipated to exhibit robust serum stability since proteases typically remain inactive outside cells, attributed to unfavorable pH conditions and the action of serum protease inhibitors. The dipeptide valine-citrulline is the most widely used enzymatic cleavage sequence, typically coupled with a self-immolative linker, p-aminobenzyl alcohol (PAB). Cleavage of an amide-linked PAB initiates a 1,6-elimination reaction, resulting in the release of carbon dioxide and the simultaneous liberation of the free drug in its parent amine form (73). Furthermore, research is currently exploring the potential of non-peptide cleavable linkers (74).

### 4.2 Bibliometric analysis

#### 4.2.1 The analysis of annual distribution of publications and citations

The distribution of the annual publications and citations reveals an unequivocally ascending trend (**Figure 1A**). The United States FDA approved the ADCs EV and SG and EV combined with pembrolizumab for the management of advanced UC in 2019. 2020 saw a substantial surge in the publications count, escalating from 28 in 2019 to 51. This upward trajectory persisted, with publication counts rising to 82 in 2021 and reaching 107 in 2022. Concurrently, the frequency of citations has been exponentially increasing since 2018, underscoring both the growing importance and promising future of ADCs in the therapeutic landscape of UC.

#### 4.2.2 The analysis of nations and institution

The scientometric analysis of nations and institutions can contribute to knowing which country and institution make the best contributions in this field. Correspondingly, data indicates that cancer incidence rates positively correlate with the Human Development Index (HDI), with male cancer mortality rates in higher HDI countries being twice as elevated compared to lower HDI nations (1). In the United States, a country with a high HDI, it was estimated that 83,730 new cases and 17,200 deaths attributed to bladder cancer occurred in 2021 (75). As the second leading cause of death, cancer has got considerable attention from the United States (75). France, with its sophisticated healthcare infrastructure, has significantly influenced the European Association of Urology Guidelines across various UC types. As shown in **Figure 1B** and **Table 1**, the USA (n=264) and France (n=61) are the countries with the most publications, Mem Sloan Kettering Canc Ctr(n=63) and Univ Washington(n=43) from USA rank the top one and two in the list of published articles, respectively. Of the top 5 institutions, there are 4 institutions belonging to the USA and 1 institution belonging to Japan. With advanced medical resources and superb scientific prowess, the USA has made great contributions to the field of ADCs in UC. As the second largest prescription drug pharmaceutical enterprise in Japan, Astellas Pharma Inc is the funding agency of many studies into therapies of UC, promoting the progression of the new therapy ADCs in UC.

#### 4.2.3 The analysis of references

Visual analysis of co-cited references provides insight into thematic similarities across articles and delineates emergent trends within the field. The most highly cited document is titled “Enfortumab Vedotin in Previously Treated Advanced Urothelial Carcinoma”. It confirms that, compared with standard chemotherapy, EV significantly prolongs survival of locally advanced or metastatic UC patients who were administered with platinum therapy and PD-1 or PD-L1 inhibitors previously. The second one, “Pivotal Trial of Enfortumab Vedotin in Urothelial Carcinoma After Platinum and Anti-Programmed Death 1/Programmed Death Ligand 1 Therapy”, substantiates a favorable clinical response rate coupled with a tolerable safety profile for patients with locally advanced or metastatic urothelial carcinoma (UC) who have previously undergone treatment with platinum-based chemotherapy and anti-PD-1/L1 immunotherapies. The third one is “Trastuzumab duocarmazine in locally advanced and metastatic solid tumours and HER2-expressing breast cancer: a phase 1 dose-escalation and dose-expansion study”revealing that Trastuzumab duocarmazine demonstrates significant clinical efficacy in extensively treated patients with HER2-expressing metastatic cancers, including those resistant to HER2-positive trastuzumab emtansine and with HER2-low breast cancer, while maintaining a manageable safety profile. From **Table 2**, we can see that articles concerning ADCs in UC have been published in recent years, and are cited quite frequently suggesting that ADCs in UC represent a focal point of contemporary research with substantial prospects for future advancements.

### 4.3 Classic ADCs in UC

#### 4.3.1 Anti-nectin-4 ADC

In April 2019, the US FDA granted approval for EV as a therapeutic agent for patients with locally advanced or metastatic UC who have demonstrated resistance to platinum-based therapies and immune checkpoint inhibitors. EV is an ADC comprising a humanized IgG1 antibody targeting nectin-4, the potent microtubule-disrupting agent MMAE and a protease-cleavable linker. After binding to nectin-4, it is internalized and degraded by endocytosis and releases MMAE. The free MMAE interferes with the intracellular microtubule architecture inducing cell cycle arrest and subsequent apoptosis, thereby inhibiting tumor growth (76). Nectin-4 is an immunoglobulin-like adhesion molecule which can mediate calcium-independent cellular adhesion at adherens junctions (77). Its transcript is minimally exhibited in various healthy tissues while markedly upregulated in many cancer types, covering breast, pancreatic, gastric, lung, and bladder cancers, thereby rendering it an optimal therapeutic target (77–82). During the process of UC metastasis and diffusion, the expression of nectin-4 is often reduced, which is related to the resistance of EV (83).

EV-101 is a Phase I clinical trial primarily assessing the safety and pharmacokinetics of EV, and the secondary focus is its antitumor efficacy (84). The study enrolled 155 mUC patients previously undergoing either platinum-based chemotherapy or at least three lines of prior treatment. Of the 112 mUC patients treated with single-agent EV, the ORR was 43%, the median duration of response (DOR) was 7.4 months, the OS was 12.3 months, and the one-year OS rate stood at 51.8%.

EV-201 is a Phase II, single-arm trial encompassing two cohorts, designed to evaluate the efficacy and safety of EV in patients with platinum chemotherapy and anti–PD-1/L1 previously treated, locally advanced or metastatic UC (85). The study comprised patients who had previously undergone treatment with both platinum-based chemotherapy and anti-PD-1/L1 agents. EV was administered to 125 patients. The ORR stood at 44%, inclusive of a 12% complete response (CR). A median follow-up duration of 10.2 months was observed, with a median DOR of 7.6 months. Cohort 2 encompassed patients who had exclusively received anti–PD-1/L1 therapy (86). The study reported a confirmed ORR of 52%, complemented by a CR rate of 20%. Furthermore, the median DOR was observed to be 10.9 months, with a median follow-up period of 13.4 months and a median PFS of 5.8 months, showing great efficacy. The promising results have promoted the FDA’s accelerated approval of EV.

EV-301 is a global, open-label, Phase III trial designed to assess the therapeutic effect of EV in comparison to conventional chemotherapy regimens (docetaxel, paclitaxel, or vinflunine) in patients presenting with locally advanced or metastatic UC (10). In these patients, disease progression occurred subsequent to platinum-based chemotherapy and during or after treatment with anti-PD-1 or PD-L1 inhibitors. The 608 patients were randomized in a ratio of 1:1 to receive EV or chemotherapy. The OS in EV group was superior to that of the chemotherapy group (12.88 vs. 8.97 months), as was the PFS (5.55 vs. 3.71 months). On July 9, 2021, the FDA approved EV for adult patients with locally advanced or metastatic urothelial cancer who have previously received a programmed death receptor-1 (PD-1) or programmed death-ligand (PD-L1) inhibitor and platinum-containing chemotherapy, or are ineligible for cisplatin-containing chemotherapy and have previously received one or more prior lines of therapy.

EV demonstrated a significant advantage in prolonging survival and enhancing quality of life relative to standard chemotherapy. Given that Nectin-4 is ubiquitously expressed across multiple organs, further research is warranted to explore its heterogeneity as a means of minimizing adverse effects.

#### 4.3.2 Anti- trop-2-ADC

Sacituzumab Govitecan(SG) is an ADC comprising the hRS7 IgG1κ monoclonal antibody conjugated to the topoisomerase I inhibitor SN-38 via a cleavable CL2A linker. The hRS7 IgG1κ, targeting Trop-2, is a transmembrane glycoprotein implicated in a variety of cellular functions, including proliferation, migration and survival of both stem and tumor cells (87–91). Numerous studies have corroborated that Trop-2 is overexpressed in a series of malignancies, such as upper tract UC, while maintaining low expression in normal tissues—thereby establishing it as an optimal therapeutic target (92–96). SN-38 is a semi-synthetic derivative of camptothecin that inhibits the nuclear topoisomerase-I (Topo-I) enzyme, thereby inducing double-stranded DNA breaks and subsequent cellular apoptosis (97). In 2021, SG received accelerated approval for the management of patients with locally advanced or metastatic UC who had previously been administered platinum-based chemotherapy and either a PD-1 or PD-L1 inhibitor.

IMMU-132-01 is a Phase I/II basket study evaluating the clinical efficacy of intravenous SG in advanced solid tumors, including mUC (98). The ORR was 31% (14/45) encompassing 2 CR and 12 partial responses (PR). The median DOR stood at 12.6 months, while the median PFS and OS were 7.3 and 18.9 months, respectively. This study revealed that SG exhibited notable clinical efficacy in the management of relapsed or refractory mUC, inclusive of patients who had prior treatment with checkpoint inhibitors (CPIs) as well as those with visceral disease involvement.

TROPHY-U-01 was a multicohort, open-label, Phase II study assessing the therapeutic effect of SG in locally advanced or unresectable or metastatic UC (11). Cohort 1 included 113 locally advanced or unresectable or mUC patients who experienced disease progression following prior platinum-based combination chemotherapy (PLT) and CPIs. With a median follow-up of 9.1 months, the ORR was 27% while 77% of the cohort experienced a reduction in measurable disease. Median DOR was 7.2 months. Median PFS and OS were 5.4 months and 10.9 months, respectively. SG has considerable efficacy relative to historical controls in the treatment of mUC that has progressed following both prior platinum-based combination chemotherapy (PLT) and checkpoint inhibitors (CPI). The outcomes from Cohort 1 of the TROPHY-U-01 trial substantiated the expedited Fast Track designation and subsequent accelerated FDA approval for the utilization of SG in the management of mUC that has previously undergone treatment with platinum-based chemotherapy and CPI.

#### 4.3.3 Anti-HER-2 ADC

Human epidermal growth factor receptor 2 (HER-2) is a growth-promoting tyrosine kinase receptor, mediating tumor proliferation, invasion and metastasis by RAS/RAF/MAPK, PI3K/Akt and other signaling pathways (99). With an acceptable safety profile, targeted therapy against HER-2 significantly enhances OS and PFS of patients with advanced HER2-positive breast cancer or gastric cancer (100–102). HER-2 is notably overexpressed in UC and exhibits a degree of heterogeneity (103). Consequently, HER-2 emerges as a promising emergent therapeutic target for UC. Recent studies have shown that HER2 overexpression is often inconsistent between primary and metastatic urothelial carcinoma and is associated with intratumoral HER2 heterogeneity (104). In HER2 positive primary lesions, the loss of HER2 overexpression in 55% (5/9) of metastatic lesions was associated with the presence of HER2 intratumoral heterogeneity in primary lesions. Several ADCs aimed at HER-2 are currently undergoing clinical trials and exhibit substantial therapeutic potential.

Disitamab Vedotin (RC48) comprises a humanized monoclonal antibody specific for HER-2, conjugated to MMAE through a cleavable linker (105). A Phase II, open-label, multicenter, single-arm study (RC48-C005) was conducted to assess the safety and efficacy of RC48 in patients with HER2-positive UC, manifesting either as locally advanced or metastatic UC, who demonstrated refractoriness to standard therapeutic regimens (106). The study demonstrated a median PFS duration of 6.9 months and an ORR of 51.2%, accompanied by an OS duration of 13.9 months. The study shows a promising efficacy of RC48 in patients with HER2 positive locally advanced or mUC who had been refractory to one or more line of systemic chemotherapy. The RC48-C009 trial was initiated to rigorously evaluate the therapeutic efficacy of RC48 in mUC patients exhibiting HER2 overexpression who failed with the treatment of platinum, gemcitabine and taxane before (107). Among all the enrolled patients, the cORR was 46.9% and the median PFS was 4.3 months with a median OS of 14.8 months. Subgroup analysis showed ORRs of 55.6%, 50.0%, and 30.8% in patients receiving one, two, and three or more lines of treatment, respectively, indicating a superior therapeutic efficacy and an enhanced benefit-risk profile relative to the data from the RC48-C005 study. RC48-C011 is a single-arm Phase II study evaluating the therapeutic effect and safety of RC48 in local advanced or metastatic UC patients with HER2-negative (IHC 0 or 1+) (108). For overall patients, disease control rate was 94.7%, the ORR was 26.3%, the median PFS was 5.5 months and the median OS was 16.4 months. For patients with IHC 1+, the ORR was 38%. The Phase III clinical trial, named RC48-C016, is actively enrolling treatment-naïve patients with HER2-overexpressing, locally advanced or metastatic UC to evaluate the comparative efficacy of RC48 and JS001 against standard chemotherapy regimens. RC48-C016 may bring new options for the first-line management of UC. Several additional trials concerning the combination of RC48 are ongoing.

Trastuzumab Emtansine (T-DM1) is composed of Trastuzumab, a stable thioether linker MCC and a microtubule inhibitor DM1. In preclinical models, T-DM1 demonstrated robust anti-tumor efficacy in bladder cancer cells overexpressing HER-2 (109). The KAMELEON, a Phase II study, aims to evaluate the optimal tumor response subsequent to T-DM1 administration in patients manifesting HER2-overexpressed solid neoplasms (110). However, due to insufficient patient enrollment, the study was prematurely terminated and primary endpoints were not achieved. Existing data indicated that the urothelial bladder cancer cohort presented an ORR of 38.5%, a median DOR of 3.38 months, and a median OS of 7.03 months, without complete responses.

Trastuzumab deruxtecan (T-DXd, DS-8201a) is formulated with trastuzumab and an enzymolytic peptide linker (111). The ongoing DS8201-A-U105, a Phase Ib trial, investigated T-DXd combined with nivolumab in patients with HER2-positive UC. Preliminary analysis suggested an ORR of 36.7%, a median DOR of 13.1 months, a median PFS of 6.9 months, and a median OS of 11.0 months, demonstrating noteworthy antitumor efficacy in UC with elevated HER2 expression (112).

A multitude of clinical trials are currently underway concerning HER2 in solid tumor including UC. RC48-C013(NCT04280341) is a Phase I study conducted to assess the safety, efficacy, survivability and pharmacokinetics of RC48 plus JS001 in HER2 positive advanced solid tumors including UC. KlusPharma(NCT03602079) is a Phase I/II first-in-human trial investigating the efficacy of A166 in patients with HER2-expressing malignancies who have either experienced disease progression or demonstrated resistance to existing standard-of-care treatments. SGNTUC-019(NCT04579380) is a Phase II basket study assessing Tucatinib plus Trastuzumab in locally advanced unresectable or metastatic solid tumors with HER2 expression. JZP712-201(NCT05126433) aims to evaluate the safety and therapeutic effect of lurbinectedin monotherapy in subjects with advanced or metastatic solid tumors.

#### 4.3.4 Anti-TF-ADC

Tissue Factor (TF) serves a pivotal role in the extrinsic coagulation pathway. Tisotumab Vedotin(TV) is an ADC targeting TF and consists of MMAE, a human monoclonal antibody TF-011 and a protease-cleavable valinecitrulline linke (113). Tisotumab vedotin has already showed clinically significant and durable tumor-suppressing effects in cervical carcinoma and has received authorization from the FDA for the management of some patients with advanced cervical carcinoma (114). Numerous studies have indicated aberrant TF expression in various malignancies (113, 115). Therefore, TF has great potential to be a novel therapeutic target in UC.

InnovaTV 201 is a Phase I/II, open-label study establishing the capacity for endurance of Tisotumab vedotin in various solid tumors (116). Of all patients, the ORR was 15.6%, of which bladder cancer and cervical cancer have similar ORR (26.7% vs 26.5%). Among responders, the median DOR was 5.7 months and median PFS was 3.0 months. GCT1015-03(NCT03245736) is a Phase 2 study exploring the therapeutic effectiveness and tolerability of continuous treatment with Tisotumab Vedotin in solid tumors with TF expression. Preliminary results reveal a partial response rate (PRR) of 40.0% and a progressive disease rate of 20%.

#### 4.3.5 Anti-EpCAM-ADC

Epithelial cell adhesion molecule (EpCAM) is an adhesion glycoprotein implicated in the modulation of cellular proliferation, differentiation, and adhesion in epithelial malignancies (117). Due to its pervasive expression, EpCAM is currently employed in local bladder perfusion administration. Elevated EpCAM expression is observed in a diverse spectrum of neoplasms, UC included, and is correlated with advanced stage, elevated histological grade, and diminished OS in patients with BC (118, 119). Urinary EpCAM levels have been identified as robust indicators of bladder cancer-specific survival (120). Thererfore, EpCAM is a promising target with great therapeutic potential in UC. Oportuzumab Monatox(OM) is an ADC targeting EpCAM which is composed of Pseudomonas exotoxin ETA252-608 and humanized single-chain variable fragment. In urinary system, OM is principally utilized for the management of non-muscle-invasive bladder cancer(NMIBC) resistant to Bacille Calmette-Guérin (BCG) treatment.

A Phase I study confirmed that OM has an antitumor effect and great tolerance in NMIBC patients with who are refractory or incompatible to BCG (121). A Phase II trial has been initiated to evaluate the therapeutic efficacy and tolerability of intravesical OM in patients with UC presenting with carcinoma *in situ* of the bladder (122). 46 patients were divided into 2 cohorts. Cohort 1 and cohort 2 received intravesical instillation of 30 mg OM with 6 or 12 weeks as an induction cycle. In the following, patients received up to 3 maintenance cycles, in which patients underwent a regimen of three weekly treatments, administered quarterly. At the 3-month evaluation, the complete response rate in cohort 1(n=9,41%) was higher than that in cohort 2(n=9,39%). In patients who got a CR, the duration to recur in cohort 1(274days) was shorter than that in cohort 2(408days). These results demonstrate that OM has great clinical benefit in patients with carcinoma *in situ* of the bladder that is refractory to BCG treatment.

#### 4.3.6 Anti-SLITRK6-ADC

SLITRK6, a constituent of the SLITRK neuronal transmembrane protein family, serves a crucial function in cellular adhesion and differentiation. Additionally, it modulates the migratory and invasive behaviors of cancer cells (123). A study has demonstrated that both upper tract urothelial carcinoma (UTUC) and urinary bladder urothelial carcinoma (UBUC) have high SLITRK6 expression, rendering it a compelling therapeutic target for the management of UC (124).

Sirtratumab vedotin (ASG15-ME) is an ADC consisting of MMAE, a humanized γ-2 antibody and a protease cleavable linker (125). NCT01963052 is a Phase I study to evaluate AGS15E in patients with mUC who have been refractory to at least one previous chemotherapy regimen. The study enrolled 93 participants and stratified them into 4 parts. The study is ongoing.

### 4.4 Combination therapy based on ADCs

Though ADCs present unique advantages in UC, the monotherapy of ADCs has not yet achieved the ideal therapeutic effects. Therefore, the focus of recent research has increasingly shifted towards the development of combination therapies based on ADCs. Ensuring safety, more effective and safe combination therapies should be explored in the future, which could enhance therapeutic efficacy and minimize treatment-related discomfort.

#### 4.4.1 ADCs combined with ICIs.

ADCs exert cytotoxic effects on tumor cells, facilitating the release of cancer antigens that subsequently activate immune responses and augment antigen-presenting cell function. Immune checkpoint inhibitors (ICIs) modulate the immune response by regulating cytokines, enzymes, and T lymphocytes involved in immunomodulatory processes, resulting in regulating immunosuppression in the tumor microenvironment. ICIs are utilized as first-line therapy in patients contraindicated for platinum-based combination regimens or as second-line therapeutic intervention for mUC. Theoretically, ADCs and ICIs exhibit synergistic antitumor activity. Further studies on the mouse model showed that the combination of PD1 inhibitors and blentuximab could increase tumor shrinkage, confirming the potential synergistic effect of the two drugs (126).

##### 4.4.1.1 EV combined with ICIs

###### 4.4.1.1.1 EV combined with pembrolizumab

In Apr. 2023, enfortumab vedotin (EV) plus pembrolizumab receieved accelerated approved by FDA for patients with advanced urothelial carcinoma who are cisplatin-ineligible. Subsequently, in December 2023, this combination received FDA approval for the treatment of patients with locally advanced or metastatic urothelial cancer.

EV-103 is a Phase I/II study determining the durability of EV combined with other anticancer therapies in mUC. One cohort enrolled 45 patients with 1L cis-ineligible and treated them with EV plus pembrolizumab. Following a median of 9 cycles, the CR rate was 15.6% and the ORR amounted to 73.3%. The median PFS was observed to be 12.7 months, with a 12-month PFS rate of 55.0%. Concurrently, the median OS spanned 26.1 months, accompanied by an 83.4% rate of OS at 12 months. The outcomes from this cohort underscored the potential efficacy, durability, and manageable safety profile associated with the EV and pembrolizumab regimen in mUC (127, 128). The favorable results of the EV-103 study expedited the approval of enfortumab vedotin (EV) in combination with pembrolizumab for the treatment of patients with advanced urothelial carcinoma who are ineligible for cisplatin therapy.

The EV-302 trial, an open-label, randomized study, involved 886 patients with locally advanced or metastatic urothelial carcinoma who had not received prior systemic therapy for advanced disease. Participants were allocated in a 1:1 ratio to receive either enfortumab vedotin-ejfv combined with pembrolizumab or platinum-based chemotherapy (gemcitabine plus cisplatin or carboplatin). Results indicated that the enfortumab vedotin-ejfv and pembrolizumab cohort achieved a higher median OS of 31.5 months compared to 16.1 months and a higher median PFS of 12.5 months compared to 6.3 months in the platinum-based chemotherapy group (8). These promising outcomes led to the approval of enfortumab vedotin plus pembrolizumab for patients with locally advanced or metastatic urothelial carcinoma.

EV-304 is a Phase III study evaluating the therapeutic effect and tolerability of perioperative EV plus pembrolizumab as compared to neoadjuvant gemcitabine/cisplatin chemotherapy in cisplatin-eligible participants with MIBC (129). The study is ongoing.

###### 4.4.1.1.2 EV combined with Durvalumab and Tremelimumab

VOLGA, another Phase III, randomized, international investigation seeks to assess the therapeutic effect and tolerability of various drug combinations (Durvalumab + Tremelimumab +EV vs. Durvalumab + EV) in cisplatin-ineligible or cisplatin-refusing patients undergoing radical cystectomy for MIBC; enrollment is currently in progress (130).

##### 4.4.1.2 RC48 combined with ICIs

RC48-C005 demonstrated a median PFS of 6.9 months and an OS of 13.9 months, with an ORR of 51.2%, showing a promising efficacy of RC48 among patients diagnosed with HER2 positive locally advanced or mUC who had experienced treatment failure following one or more lines of systemic chemotherapy therapies (106). RC48-C014 is a phase Ib/II trial assessing the safety and pharmacokinetics of RC48 plus Torialimab in locally advanced or metastatic UC. By Nov. 2022, for overall patients, the confirm ORR was 73.2%, the CR rate was 9.8%, median PFS was 9.2 months and the OS of 2 years was 63.2% (131). The ORR in HER2 IHC 2/3+,IHC 1+ and IHC 0 were 83.3%,64.3% and 33.3%, respectively. The ORR was 61.5% in PD-L1-positive cases and 78.6% in PD-L1-negative cases. RC48-C016, a Phase III multicenter trial, is designed to evaluate the therapeutic effect of RC48 plus Torialimab compared to standalone chemotherapy in untreated participants with unresectable, locally advanced, or metastatic UC exhibiting HER2 expression. RC48-C016 is recruiting.

There is also a multicenter, real-world investigation enrolling 36 patients with locally advanced or metastatic UC to assess the therapeutic effectiveness of RC48, either alone or combined with PD-1 antibodies such as toripalimab, tislelizumab, pembrolizumab, envafolimab, and sintilimab (132). Nearly half (47.2%) of the participants were administered with RC48 after second-line therapy. The median PFS was 5.4 months, while the median OS remained undefined. Both the 1-year PFS and 1-year OS were 15.5%. The 6-month PFS rate was 38.8%. Notably, the median PFS was 8.5 months in patients receiving the combination therapy, compared to 5.4 months in those treated with RC48 monotherapy. These results support the potential utility of RC48, either as a standalone treatment or in conjunction with immunotherapy, in managing patients diagnosed with locally advanced or metastatic UC, irrespective of renal function status.

##### 4.4.1.3 SG combined with ICIs

###### 4.4.1.3.1 SG combined with Pembrolizumab

SURE-02, a perioperative Phase 2, single-cohort study is designed to evaluate the therapeutic efficiency of perioperative Pembrolizumab plus SG in patients diagnosed with muscle-invasive BC who are ineligible for or decline cisplatin-based chemotherapy. The study is ongoing.

###### 4.4.1.3.1 SG combined with Ipilimumab plus Nivolumab

NCT04863885 is Phase I/II study assessing the first-line therapeutic efficiency of Ipilimumab plus Nivolumab (IPI-NIVO) in combination with SG in cisplatin-ineligible mUC. The study is ongoing.

The combination of ADCs and ICIs expanded the scope of patients and showed promising therapeutic effect. Many clinical trials related to the combination were enrolling or ongoing and are worth looking forward to.

#### 4.4.2 ADCs combined with chemotherapy

For patients diagnosed with metastatic urothelial carcinoma (mUC), the standard first-line therapeutic regimen comprises cisplatin-based chemotherapy, utilizing either gemcitabine/cisplatin or dose-dense methotrexate, vinblastine, doxorubicin, and cisplatin (dd-MVAC) (133). Previous studies have reported an OS range of 12-14 months for cisplatin-containing regimens, with OS rates for MVAC and gemcitabine/cisplatin (GC) documented at 14.8 and 13.8 months (134, 135). As emerging anti-tumor drugs, ADCs offer the advantage of targeted payload delivery to tumor cells, thereby mitigating systemic side effects. With longer median OS and PFS and lower mortality compared with chemotherapy in EV-301, FDA has approved EV as a second-line therapeutic option for patients with progressive disease subsequent to platinum-based chemotherapy and anti-PD-(L)1 therapy, as well as for those who are cisplatin-ineligible (10). Additionally, SG secured FDA approval for mUC treatment in April 2021, based on promising Phase I and Phase II data (11, 136). ADCs and chemotherapy provided a new combination therapy for mUC patients, and the clinal trials were ongoing.

EV-103(NCT03288545) is a Phase I/II clinical trial assessing the therapeutic effect of EV alone or with other treatments in UC. The study has enrolled 348 patients, stratified into 10 distinct cohorts, each receiving EV monotherapy or combined with pembrolizumab, cisplatin, carboplatin, or gemcitabine. Cohort D focuses on the first-line therapy impact of EV combined with cisplatin in patients eligible for cisplatin-based chemotherapy, without previous treatment for locally advanced or metastatic UC (la/mUC), and who have not received adjuvant/neoadjuvant platinum-based therapy within the preceding 12 months. Cohort E evaluates the first-line treatment efficacy of EV and carboplatin in patients who are ineligible for cisplatin but suitable for carboplatin, with similar treatment history requirements as Cohort D. Cohort F investigates the effects of EV combined with gemcitabine for first-line and second-line therapy in patients who are intolerant for platinum-based chemotherapy, but suitable for gemcitabine, or have manifested disease advancement post at least one prior la/mUC treatment. The trial is ongoing. NCT05723991 is a multicenter, Phase II clinical trial designed to evaluate the therapeutic effect and safety of RC48 combined with gemcitabine in preoperative neoadjuvant treatment of MIBC. The study enrolled 36 participants with MIBC expressing HER2 who were not suitable for cisplatin chemotherapy. The study is in progression and is estimated to be completed in Sep.2025.

#### 4.4.3 ADCs combined with ADCs

With high targeting efficiency and high activity in tumor tissues, ADCs have emerged as second-line and even first-line treatment drugs for cancer patients. Clinical trials investigating ADCs in UC have proliferated in recent years. Combinations of ADCs with well-tolerated safety profiles, efficacious outcomes, and non-synergistic adverse effects appear promising as novel therapeutic strategies in UC. Experiments have shown that ADC has a non-overlapping resistance mechanism (137). After prolonged exposure to EV, cells can downregulate NECTIN4, leading to EV resistance, but retaining TROP2 expression and maintaining sensitivity to SG, which shows ADCs combined with ADCs have great therapeutic potential. EV and SG have already got FDA approval for the treatment of some UC patients and existing research substantiates their safety and efficacy profiles (10, 11, 84, 85, 138). A Phase I trial (NCT04724018) was designed to assess the safety and therapeutic effect of EV plus SG and assess safe dose profiles of SG and EV in mUC patients experiencing disease progression subsequent to platinum-based chemotherapeutic intervention and PD-1/PD-L1 blockade (139).

#### 4.4.4 ADCs combined with targeted therapy

Targeted therapeutics inhibit neoplastic proliferation by interacting with specific molecular determinants critical for oncogenesis and tumoral expansion. Numerous patients exhibit genetic alterations in kinases and key cellular mechanisms (140). Currently, the sole validated alterations amenable to therapeutic intervention are activating mutations or fusions involving FGFR2 and FGFR3. As a pan-FGFR inhibitor, erdafitinib had received approval by FDA for la/m UC patients who have progression subsequent to platinum-based chemotherapeutic intervention and who are vulnerable FGFR3 or FGFR2 genomic alterations. Both antibody-drug conjugates (ADCs) and targeted therapeutics demonstrate the capacity to attenuate tumor cell proliferation, and multiple ongoing clinical trials are investigating the prospective synergistic effects of the combined modalities.

The optimal dosage, prospective therapeutic advantages, and associated adverse events of Erdafitinib plus EV in patients with metastatic bladder cancer and FGFR2/3 genes alterations were studied in a phase Ib clinical trial (NCT04963153). NCT04878029 was a phase I/Ib trial finding out the optimal dosage, prospective therapeutic advantages, and associated adverse events of Cabozantinib combined with EV in la/m UC patients. Patients receive Cabozantinib orally once daily on days 1-28 and EV intravenously on days 1, 8, and 15. Treatment cycles are reiterated on a 28-day interval, contingent on the absence of either disease advancement or prohibitive toxicity. More clinical trials concerning the combination of ADCs and targeted drugs are ongoing.

## 5 Conclusions

Both the scientometric analysis of the 475 publications and the clinical trial analysis corroborate the rapid advancement of ADCs in UC. Through clinical trial analysis, we could find some further information on the research trends in this field. Many clinical trials related to ADCs with therapeutic potential are ongoing, especially HER-2. ADCs have transitioned from posterior line therapy to frontline therapeutic options in advanced HER-2 positive UC, and they are expected to redefine the traditional first-line chemotherapeutic landscape for this malignancy. In the clinical trials, combinations of ADCs and other therapies are the majority, with the most common combination being ADCs and ICIs. What is more, preliminary investigations into ADC-based combination therapies are underway, particularly in the neoadjuvant and adjuvant settings for HER-2 positive UC. Future research hotspots of ADCs are anticipated to include the discovery of new therapeutic targets, the optimization of combination therapies and the personalization of treatment regimens. Additionally, elucidating drug resistance mechanisms and exploring ways to surmount such resistance are crucial for advancing the role of ADCs in the management of UC. We hope this study will help researchers understand more fully the current status and research trends of ADCs in UC.

## 6 Present state and prospective outlook

Though ADCs have made great progress in UC, challenges persist in the prospective advancement of ADCs for solid tumors.

### 6.1 Drug resistance

Drug resistance in cancer cells presents a significant challenge to cancer treatments, including ADCs. Under the pressure of treatment, cancer cells can develop mechanisms of resistance that enable their survival, leading to reduced drug sensitivity and diminished therapeutic effectiveness. The specific mechanisms underlying resistance to ADCs remain unclear, and it is hypothesized that they may be related to the individual components of the ADCs (141).

#### 6.1.1 Antigen-related resistance

Antigen-related resistance significantly impacts the efficacy of ADCs, as evidenced by various experiments. For instance, in a study involving breast cancer cell lines, repeated exposure to trastuzumab–maytansinoid ADC at IC80 concentrations resulted in acquired resistance. This resistance, induced by chronic drug treatment, was primarily mediated through increased ABCC1 protein expression or reduced Her2 antigen levels (142). Another case report highlighted the loss of CD30 expression in a patient with anaplastic large cell lymphoma following brentuximab vedotin therapy (143). The presence of antigen ligands can modulate the sensitivity of ADCs. Research indicates that certain ligands, like neuregulin, which facilitates the heterodimerization of HER2 with HER3 and HER4, may reduce the efficacy of T-DM1 (144).

#### 6.1.2 Impaired lysosomal function

Impaired lysosomal function can significantly reduce the efficacy of ADCs, especially those with non-cleavable and lysosomal protease-sensitive cleavable linkers. This is because these ADCs release their payloads within lysosomes. Research has shown that in certain resistant cells, T-DM1 accumulates in lysosomes, leading to an increased pH and disrupted proteolytic activity in these organelles (145). Additionally, the transport of cytotoxic agents from the lysosomal lumen to the cytoplasm presents another resistance mechanism. The partially decomposed metabolites of ADCs with non-cleavable linkers within lysosomes are unable to directly penetrate the lysosomal membrane to enter the cytoplasm; instead, they are transported via a specific mechanism. A study utilizing phenotype shRNA screening on CD70 resistant zeatin-based ADCs identified SLC46A3, a lysosomal membrane protein. The genetic suppression of SLC46A3 impaired the efficacy of various non-separable antibodies, including T-DM1-zeaxanthin ADCs (146).

#### 6.1.3 Drug efflux pumps

Drug efflux pumps, particularly the ATP-binding cassette (ABC) transporters, represent a common resistance mechanism in chemotherapy, facilitating the removal of therapeutic agents from the cellular cytoplasm (147). This mechanism is also relevant to ADCs, as many cytotoxic agents used in ADCs are substrates of these ABC transporters (148). A notable study investigating resistance to gemtuzumab ozogamicin (GO) demonstrated this mechanism. In the study, HL-60 cells were consistently exposed to GO, creating GO-resistant HL-60 (HL-60/GOR) cells (149). These cells exhibited strong expression of multidrug resistance 1 (MDR-1), unlike the non-resistant HL-60 cells. Interestingly, MDR-1 expression in HL-60/GOR cells decreased to trace levels upon GO withdrawal, but reintroducing GO reinstated high MDR-1 expression. This indicates that HL-60/GOR cells had developed the ability to induce MDR-1 expression in response to GO exposure. Another example in a preclinical T-DM1 resistance model showed the functional induction of MRP1. The sensitivity to T-DM1 was restored either by using an MRP1 reversal agent or through siRNA-mediated knockdown of MRP1 (142).

#### 6.1.4 Defects in internalization and trafficking pathways

ADCs require endocytic uptake through various routes, including clathrin-mediated (CME), caveolin-mediated, and clathrin–caveolin-independent endocytosis (141). A notable study identified caveolae-mediated endocytosis as a novel resistance mechanism to trastuzumab emtansine (T-DM1) (150). In this study, HER2+ cell lines were subjected to a cyclical dosing regimen of T-DM1, alternating between treatment and non-treatment periods until T-DM1-resistant populations emerged. Comparative proteomic profiling of these cells indicated an enrichment in proteins facilitating caveolae formation and endocytosis, suggesting that caveolae-mediated endocytosis of T-DM1 could be a predictive biomarker for patient response to this therapy.

To counteract drug resistance, it is essential to implement various strategies. In cases of antigen downregulation or deletion, combinations with other targeted drugs are the preferred approach (142). Exploring alternative mechanisms of ADCs can also be effective in overcoming specific resistances, which includes bi-payloads and immune-stimulating payloads. Bispecific antibodies, known for increasing tumor cell affinity and enhancing internalization efficiency, are equally effective in resistance management. For patients exhibiting endocytosis deficiency, the development of noninternalized ADCs, which release their payload directly into the tumor microenvironment for absorption by tumor cells, is a promising approach.

### 6.2 The shortage of ADCs

Besides drug resistance, manufacturing is a big challenge and is an expensive cost. ADCs are complex molecules which require specialized manufacturing processes and expertise. This could result in limitations in their production and availability, leading to a shortage of supply and expensive price. In addition, off-target toxicities pose a significant clinical concern. On one hand, some antigens could express in normal tissues; ADCs targeting the normally expressing antigens would target the normal tissues and influence the function or the growth of normal tissues. On the other hand, drug-releasing enzymes and other factors that could cleave the linker of ADCs often exist in normal tissues and ADCs would non-specifically release payloads, resulting in toxicity to normal tissues.

### 6.3 The future of ADCs

For the advancement of ADCs, several strategic solutions merit exploration. Initially, enhancing the components of ADCs could increase specificity, stability, and efficacy while mitigating toxicity. Presently, many ADC monoclonal antibodies serve merely as vehicles for cytotoxin delivery, neglecting the immunomodulatory potential inherent in antibody design. By employing antibodies with synergistic payload effects, one may achieve improved therapeutic outcomes. Developing bispecific antibodies is another idea to optimize antibodies. Moreover, to address the issue of nonspecific drug release, a comprehensive understanding of the tumor cell’s intracellular environment is essential for designing highly specific linker mechanisms. Regarding payloads, current options remain limited both in type and mechanism of action; hence, the exploration of novel anti-tumor agents acting on various targets is warranted. Diveristy should be developed in payloads, such as toxins with multiple mechanisms of action, carrying multiple loads simultaneously, immune stimulation loads, radioactive nuclides, etc.

Secondly, the emerging field of double antibody drug conjugates (DAD) represents a significant advancement in oncological therapeutics. Diverging from the 15 approved ADCs, DADs are composed of two distinct antibodies, each connected to cytotoxic drugs via specialized linkers. This dual-antibody approach enhances ADCs efficacy, particularly against targets with limited internalization. One antibody targets tumor cell-related antigens, while the other facilitates molecular internalization and degradation. This dual binding not only improves specificity but also minimizes off-target toxic effects. Additionally, by obstructing two separate signaling pathways, DADs potentially increase cellular cytotoxicity. However, their complex structure poses considerable challenges in research and development. A Phase I trial (NCT04724018) is currently assessing the safety and efficacy of an SG plus EV DAD in metastatic urothelial carcinoma, marking the first instance of combined ADC therapy in any malignancy. The trial demonstrated a promising objective response rate of 70% (151). Building on these findings, ongoing studies are exploring combinations of SG and EV, both alone and in conjunction with pembrolizumab, for urothelial carcinoma treatment.

Thirdly, non-internalized ADC is a promising direction for future research, utilizing the unique microenvironment of tumors to directly release loads outside tumor cells. Compared to internalized ADCs, non-internalized ADCs have a wider range of target selection, freeing it from dependence on high antigen expression and endocytosis, and maximizing the “bystander effect”. Fourthly, combination therapies offer promising avenues for mitigating resistance and enhancing treatment efficacy. Combining medication with different mechanisms of action can reduce the incidence of drug resistance. Simultaneously combining different drugs with similar effects can greatly increase the anti-cancer effect. At present, the combination of ADC and immunotherapy plays an important role in clinical practice and has become a new research hotspot. With the development of bispecific antibody conjugated drugs, the combination of bispecific antibody conjugated drugs and immunotherapy will also become a future research hotspot. What is more, manufacturing innovations are necessary. Ongoing research aims to simplify and optimize ADCs production methods to enhance accessibility and cost-effectiveness. Furthermore, the differences in how we approach and treat various histopathological variations represent a gap in our current knowledge and practices that has yet to be fully explored. Lastly, the future likely holds the potential for personalized medicine; utilizing genetic and molecular profiling could guide the selection of treatments tailored to individual patients, thereby reducing adverse effects.

As an evolving class of anti-tumor agents, ADCs have experienced significant advancements in recent years, with an expanding number of approvals for the treatment of various malignancies, including UC. Key areas for further enhancement include the optimization of ADCs components, the investigation of combination therapies, the research on DAD, the tailoring of individual treatments, and the refinement of manufacturing processes to broaden their therapeutic applicability and improve clinical outcomes.

### 6.4 Advantages

This study systematically analyzes the relevant literature and clinical trials of ADCs in UC, affording clinicians and investigators a comprehensive overview in this domain. This study applies various bibliometric tools to analyze relevant literature, analyzes relevant clinical trials from multiple dimensions and presents the current development status of ADCs in UC in a concise and clear manner. Through multiple analyses, we have predicted the future research hotspots and development directions in this field are exploration of new targets, combination therapy, individualized treatment and overcoming drug resistance.

### 6.5 Limitaions

Although this study provides a comprehensive review of literature and clinical trials of ADCs in UC, it is not without limitations. Firstly, the literature review may not encompass an exhaustive scope, as it relies solely on publications sourced from WoSCC and utilizes a restricted array of searching terms. Secondly, there are temporal limitations. The searching process was conducted in early 2023, precluding the inclusion of papers published subsequent to our search date, although the influence of such omissions on the overall conclusions is anticipated to be minimal. Moreover, owing to space constraints, we selectively discussed representative clinical trials, without providing an exhaustive list of all pertinent studies. Despite these limitations, this study furnishes valuable overarching insights into this field and offers direction for future research endeavors.

## Author contributions

MZ: Data curation, Formal analysis, Investigation, Methodology, Project administration, Resources, Software, Validation, Visualization, Writing – original draft, Writing – review & editing. YZ: Software, Visualization, Writing – review & editing. SC: Writing – review & editing. YL: Software, Visualization, Writing – review & editing. YX: Software, Writing – review & editing. LY: Writing – review & editing, Software. HW: Conceptualization, Funding acquisition, Supervision, Writing – review & editing. RG: Conceptualization, Funding acquisition, Supervision, Writing – review & editing.
